# The JNK Inhibitor XG-102 Protects against TNBS-Induced Colitis

**DOI:** 10.1371/journal.pone.0030985

**Published:** 2012-03-13

**Authors:** Kirstin Reinecke, Sevgi Eminel, Franziska Dierck, Wibke Roessner, Sabine Kersting, Ansgar Michael Chromik, Olga Gavrilova, Ale Laukevicience, Ivo Leuschner, Vicki Waetzig, Philip Rosenstiel, Thomas Herdegen, Christian Sina

**Affiliations:** 1 Institute of Experimental and Clinical Pharmacology, University Hospital Schleswig-Holstein, Campus Kiel, Kiel, Germany; 2 Pharmaceutical Institute, University of Kiel, Kiel, Germany; 3 Department of Visceral and General Surgery, St. Josef Hospital, Ruhr-University Bochum, Bochum, Germany; 4 Institute for Clinical Molecular Biology, University of Kiel, University Hospital Schleswig-Holstein, Kiel, Campus Kiel, Kiel, Germany; 5 Department of Physiology, Medical Academy, Lithuanian University of Health Sciences, Kaunas, Lithuania; 6 Institute of Pathology, University Hospital Schleswig-Holstein, Campus Kiel, Kiel, Germany; Institute Pasteur, France

## Abstract

The c-Jun N-terminal kinase (JNK)-inhibiting peptide D-JNKI-1, syn. XG-102 was tested for its therapeutic potential in acute inflammatory bowel disease (IBD) in mice. Rectal instillation of the chemical irritant trinitrobenzene sulfonic acid (TNBS) provoked a dramatic acute inflammation in the colon of 7–9 weeks old mice. Coincident subcutaneous application of 100 µg/kg XG-102 significantly reduced the loss of body weight, rectal bleeding and diarrhoea. After 72 h, the end of the study, the colon was removed and immuno-histochemically analysed. XG-102 significantly reduced (i) pathological changes such as ulceration or crypt deformation, (ii) immune cell pathology such as infiltration and presence of CD3- and CD68-positive cells, (iii) the production of tumor necrosis factor (TNF)-α in colon tissue cultures from TNBS-treated mice, (iv) expression of Bim, Bax, FasL, p53, and activation of caspase 3, (v) complexation of JNK2 and Bim, and (vi) expression and activation of the JNK substrate and transcription factor c-Jun. A single application of subcutaneous XG-102 was at least as effective or even better depending on the outcome parameter as the daily oral application of sulfasalazine used for treatment of IBD.

The successful and substantial reduction of the severe, TNBS-evoked intestinal damages and clinical symptoms render the JNK-inhibiting peptide XG-102 a powerful therapeutic principle of IBD.

## Introduction

Modulators of immune cells or immune responses belong to the most intensively studied and most promising drugs. This holds also true for the common diseases with inflammatory and/or auto-immune pathologies such as rheumatoid arthritis, asthma, multiple sclerosis, cancer or inflammatory bowel disease (IBD). The pathogenetic development of IBD is characterized by three causative areas such as (i) genetic susceptibility for mutations of proteins facilitating the access of microbes to the epithelium and underlying layers, (ii) luminal antigenic provocation from endogenous microbes or food allergens and (iii) environmental triggers (reviewed by [1], [2], [3], [4], [5], [6]). Many of these changes or risk factors are linked to the signalling pathways regulating both the innate and adaptive immune system. As a consequence, experimental strategies and present-day medical therapies aim at the control of immune reactions and T cell responses e.g. by aminosalicylates, corticosteroids, immunosuppressants and immunomodulators including TNF-α-blockers [7], [8].

The JNK, a group of mitogen-activated protein kinases (MAPK) family members, are crucial mediators of various pathological signalling pathways underlying IBD. JNK activity, which is increased in IBD patients [9], [10], [11], [12], sensitizes epithelial cells against bacterial components and cytokines [13], [14]. Among numerous pathological alterations, JNKs are also involved in the maturation and function of T cells, the production of cytokines and the TNF-α-induced expression of E-selectin on endothelial cells, which is critical for leukocyte adhesion and infiltration. Moreover, JNK act as transducers of endoplasmatic reticulum (ER) stress [3], [9], [11], [15], [16], [17], [18], [19]. Therefore, inhibition of JNKs emerges as a promising therapeutic principle in various inflammatory diseases including IBD [3], [20], [21], [22] and was previously shown to counteract colorectal tumorigenesis [23], [24] that occurs in the cause of chronic IBD.

So far, the development of anti-JNK therapies was limited due to the lack of appropriate inhibitors with high specificity and cell-permeability. The TAT-fused JNK-inhibiting peptide XG-102 (D-JNKI-1) meets both criteria and is a substantial advancement [25]. As demonstrated in several preclinical studies, XG-102 and similar peptidergic JNK-inhibitor could improve the outcome in several degenerative processes like hearing loss [26], cerebral ischemia [27], haemorrhage [28], retinal neovascularisation and retinal exitotoxicity [29], metabolic syndromes including diabetes [30], [31], as well as atherosclerosis [32] (reviewed by [33], [34], [35]). In clinical studies, XG-102 demonstrated therapeutic effectiveness in traumatic hearing loss [36] and uveitis [37].

In the present study, we investigated for the first time the therapeutic potential of the JNK-inhibiting peptide XG-102 to counteract the pathological features provoked by TNBS, an acute model of IBD with predominant T helper 1 (Th1)-mediated immune response [38], [39].

The single subcutaneous application of 100 µg/kg XG-102 powerfully reduced the pronounced clinical, histological and immunological alterations and lesions following acute TNBS colitis in mice. The therapeutic effectiveness of a single XG-102 application was at least as potent as the repetitively applied standard competitor and clinical drug sulfasalazine.

## Results

### Disease activity index (DAI)

#### Normal mice

**Figure 1:**
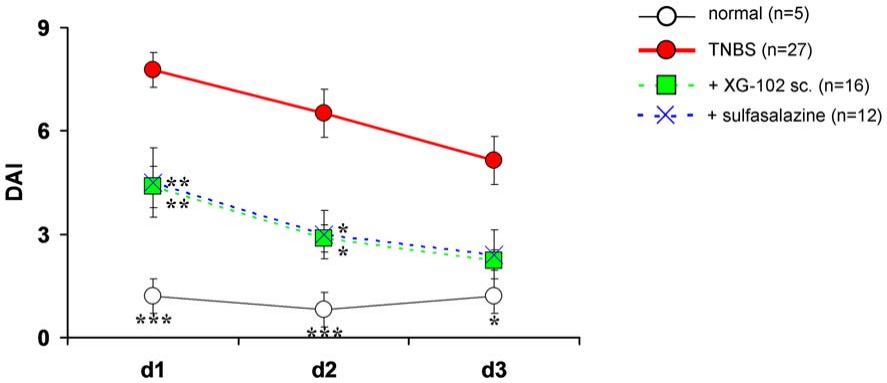
Time course of disease activity index. Time course of disease activity index (DAI) (mean+SEM) in normal mice (normal, n = 5), following rectal administration of trinitrobenzene sulfonic acid (TNBS) only (TNBS, n = 27) or treatment with XG-102 (100 µg/kg sc., n = 16) or sulfasalazine (10 mg/kg, po. daily, n = 12). *, **, *** = p<0.05, p<0.01 and p<0.001 for all groups compared with TNBS group.

Normal mice gained per average 1.7% on average of their body weight within 72 h , the hemoccult of all normal mice was negative and DAI did not surpass 1.3 (Fig. 1).

#### TNBS

After TNBS instillation, weight loss started rapidly. Within 24 h, animals lost 10.5% per average of their body weight and after 72 h, the end of the observation period, weight loss was still 9.3% compared to the body weight before the TNBS administration. TNBS provoked severe diarrhoea with blood. The initial hemoccult score was 2.5 on average after 24 h and declined to 1.1 after 72 h. The DAI reflected these changes with a maximum of 7.8 after 24 h, and it was still elevated with 5.1 after 72 h (Fig. 1).

#### Subcutanous XG-102

The sc. injection of XG-102 significantly and dose-dependently attenuated the clinical parameters. One hundred µg/kg XG-102 sc. most effectively reduced weight loss and the animals almost completely regained their initial body weight. Moreover, the hemoccult score was significantly (p<0.05, data not shown) attenuated after 24 h and was almost normal after 72 h. In consequence, XG-102 dose-dependently diminished the composed DAI by more than 50% (p<0.01) at 100 µg/kg after 24 and 48 h (p<0.05) (Fig. 1). For sc. application of 50 µg/kg XG-102 scores were all consistently better compared to solvent treatment, but did not reach significance. 10 µg/kg sc. XG-102 was not effective (data no shown).

#### Oral sulfasalazine

Daily 10 mg/kg sulfasalazine was as effective for the reductions of weight loss and DAI as single sc. 100 µg/kg XG-102 (Fig. 1).

### Gross pathology and histological analysis (H&E stain)

Macroscopical inspection of the colon immediately after the animals' death, showed a strong hyperemia, necrosis, and inflammation after TNBS administration. It was obvious that 100 sc. XG-102 or 10 mg/kg po. sulfasalazine attenuated these features.

### Tissue damage

#### Normal mice

Healthy mice displayed regular crypts with goblet cells; lymphocytes in the lamina propria were rarely detectable. Occasional lymphoid nodules were identified in the submucosa. Inflammatory infiltrates were absent. Normal crypts were linked with the underlying lamina muscularis.

#### TNBS

**Figure 2:**
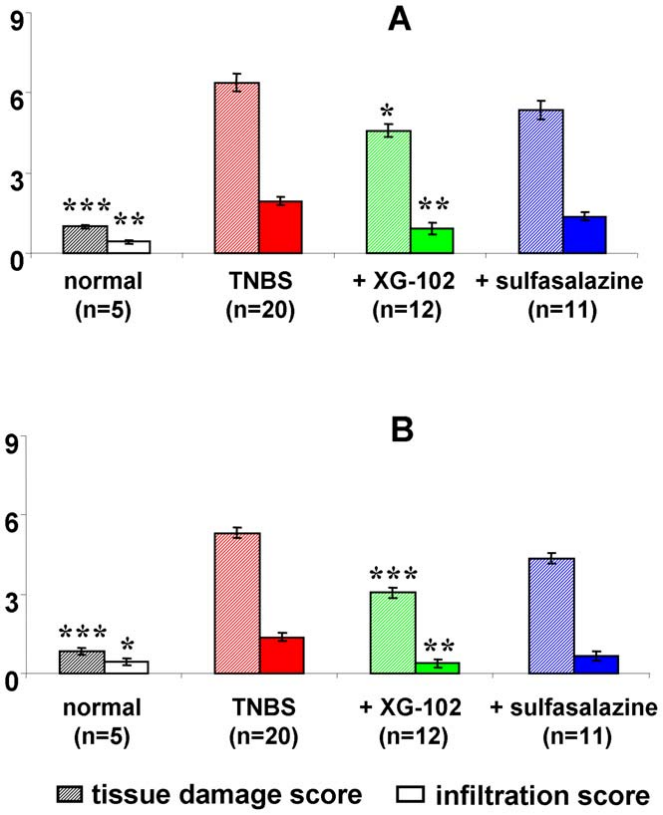
Hematoxylin and eosin (H&E) scores. Hematoxylin and eosin (H&E) scores from distal (A) and medial (B) colon. For the tissue damage score (hatched bars), the scores of ulcer, crypts and submucosa were summed-up for each individual animal, and the mean±SEM was calculated for each group. The mean±SEM of the infiltration score (grey bar) is separately shown. ***, ** = p<0.001 and p<0.01 for all groups compared with TNBS group.

The TNBS administration caused a severe transmural inflammation characterized by infiltration of inflammatory cell (Fig. 2). The inflammation was accompanied by multifocal dropouts of entire crypts and severe ulcerations, loss of goblet cells, and fibrosis. As a further characteristic change of the acute inflammation, the submucosa was enlarged by a pronounced oedema. The maximum of pathological alterations was found in the distal part of the colon, but was also present in the medial part (Fig. 2).

#### XG-102

Subcutaneous application of 100 µg/kg XG-102 significantly reduced the parameters of inflammation and tissue destruction. The total score of the three histological parameters was significantly attenuated by sc. XG-102 equally in the distal colon (p<0.01) and medial colon (p<0.001) (Fig. 2). XG-102 almost completely restored the histological appearance of the mucosa compared with the TNBS group.

#### Sulfasalazine

Sulfasalazine reduced the H&E parameters in the distal and medial colon (Fig. 2), but did not reach significance.

### Infiltration of immune cells

Besides the severe tissue damage, TNBS caused a massive infiltration of immune cells in the distal colon and to a lesser extent in the medial part. The infiltration comprised the invasion of macrophages, lymphocytes and leukocytes (Fig. 2).

Only 100 µg/kg XG-102 sc. significantly (p<0.01) lowered the infiltration score in both the distal and medial colon. The decrease following sulfasalazine did not reach significance (Fig. 2).

### CD3 and CD68 Immunofluorescence

#### Normal mice and TNBS

**Figure 3:**
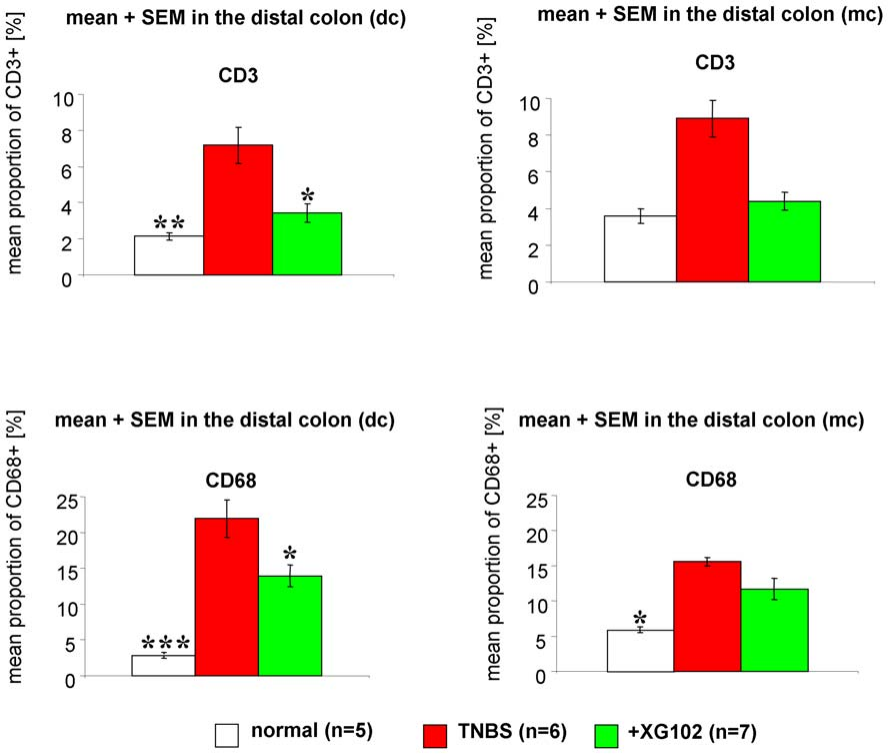
Infiltration and presence of CD3- and CD68-positive cells. The proportion (%) of CD3- or CD68-positive cells from all cells within a defined vision field in the distal and medial colon from normal mice (normal, n = 5), following rectal trinitrobenzene sulfonic acid (TNBS) administration only (TNBS, n = 6) and treatment with XG-102 (100 µg/kg sc.) (n = 7). *, ** = p<0.05 and p<0.01 compared with TNBS group.

**Figure 4:**
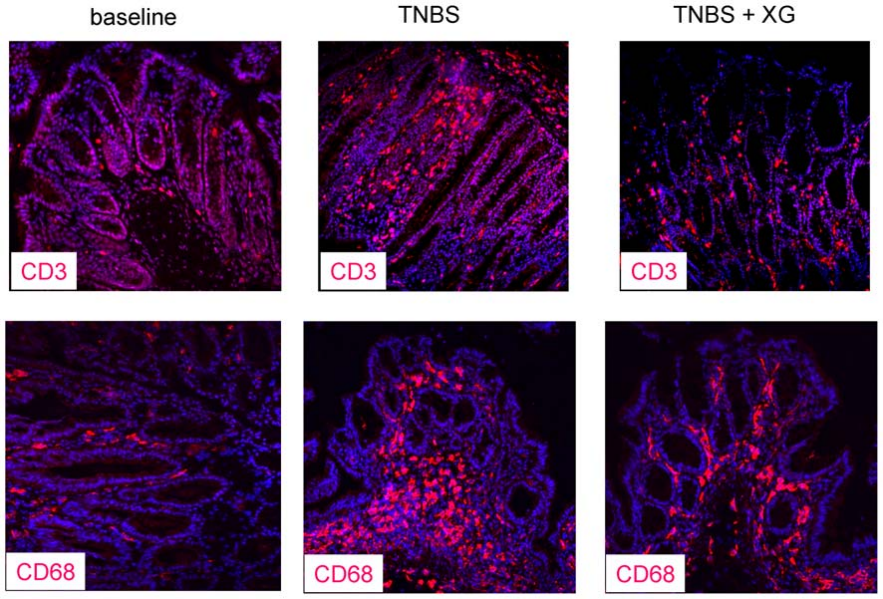
Representative CD3 and CD68 immunofluorescence. Representative CD3 (left) and CD68 (right) immunofluorescence of the distal colon from normal mice, trinitrobenzene sulfonic acid (TNBS) administration and treatment with sc. 100 µg/kg XG-102.

In normal animals, only few CD3- and CD68-positive cells were detectable (Fig. 3, 4). TNBS evoked a dramatic infiltration of CD3- and CD68-positive cells in the lamina propria, submucosa and epithelium (Fig. 3, 4).

#### XG-102

Single subcutaneous application of 100 µg/kg XG-102 significantly (p<0.05) diminished the numbers of CD3- and CD68- positive cells in the distal colon (Fig. 3). The CD3/CD68-signals were attenuated in all layers of the distal colon, i.e. epithelium, lamina propria and submucosa (Fig. 4). In the medial colon, the reduction of CD3-positive cells reached almost significance.

### Production of TNF-α

**Figure 5:**
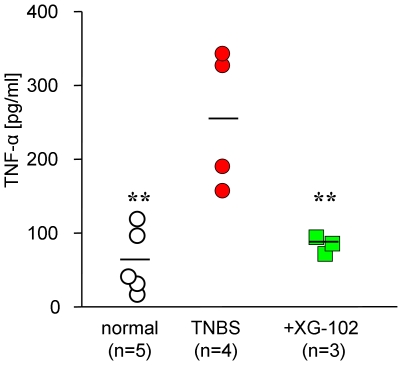
Production of TNF-α. TNF-α release (pg/ml) into the supernatant of organic colon culture from normal mice, following trinitrobenzene sulfonic acid (TNBS) only, and treatment with sc. 100 µg/kg XG-102. ** = p<0.01 for all groups compared with TNBS group.

TNF-α release was measured in organic colon cultures 24 h after the removal of the colon. TNBS provoked a dramatic rise in TNF-α concentration to 261±47 pg/ml compared to normal mice (67±20 pg/ml). Treatment with XG-102 (100 µg/kg sc.) significantly (p<0.01) reduced the TNF-α production (89±6 pg/ml) to concentration levels of normal mice (Fig. 5).

### Markers of Apoptosis

**Figure 6:**
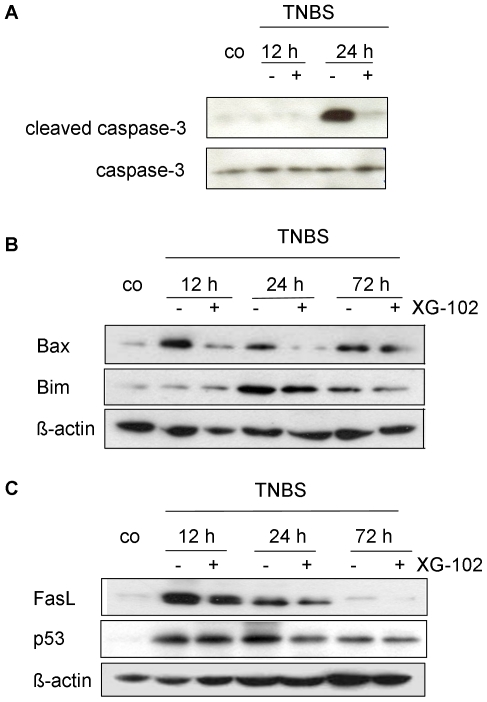
Apoptosis. Western blot analysis of (A) caspase-3 and cleaved caspase-3, (B) Bax and Bim, and (C) FasL and p53 from colon extracts of untreated controls (co) 12 h, 24 h and 72 h following trinitrobenzene sulfonic acid (TNBS) administration without or with XG-102 (100 µg/kg sc.). These blots are representative of 3 independent experiments.

Inflammation in the gut causes apoptotic cell death in intestinal mucosa [5], [7], [40]. To investigate the potential of XG-102 to attenuate TNBS-induced apoptosis, Western blot analysis of apoptotic proteins were performed with lysates from colon tissue 12 h, 24 h and 72 h after TNBS application. The levels of cleaved i.e. activated caspase-3 activity were substantially enhanced after 24 h compared to normal mice (Fig. 6A). XG-102 (100 µg/kg sc.) almost completely abrogated the signal of caspase-3 activity, whereas the presence of total uncleaved caspase-3 remained unchanged (Fig. 6A). Immunoreactivity of cleaved caspase-3 was localized in the deranged epithelium (data not shown). Protein expression of Bax and Bim, regulators of programmed cell death, were increased with maximal levels after 12 h and 24 h, respectively (Fig. 6B). XG-102 lowered these expressions at all time points investigated (Fig. 6B). Similarly, XG-102 attenuated the expression levels of FasL and p 53 (Fig. 6C).

**Figure 7:**
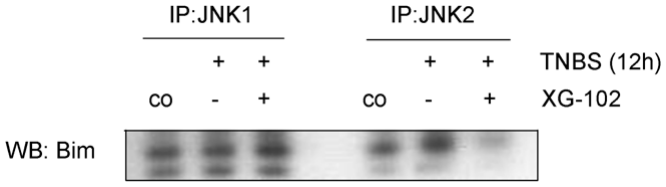
JNK2-Bim co-precipitation. JNK1 and JNK2 immunoprecipitates (IP) from colon tissue homogenates were analyzed by Western blotting with an anti-Bim antibody. Pounceau staining demonstrated equal loading (data not shown).

The activation of Bim depends on its physical interaction with JNK [41] but this issue has not been addressed so far in intestinal tissues. Here we provide evidence that both, JNK1 and JNK2 can be seen in Bim positive protein complexes, but it is JNK2 only that is increased in this protein pool from whole cell lysates (Fig. 7). XG-102 does not only prevent this up regulation but almost completely abolishes the Bim-JNK2 signal.

Summarizing these data that JNK are strong inducers of TNBS-induced apoptosis. The JNK inhibitor XG-102 substantially reduces various apoptotic features.

### Expression and activation of c-Jun

**Figure 8:**
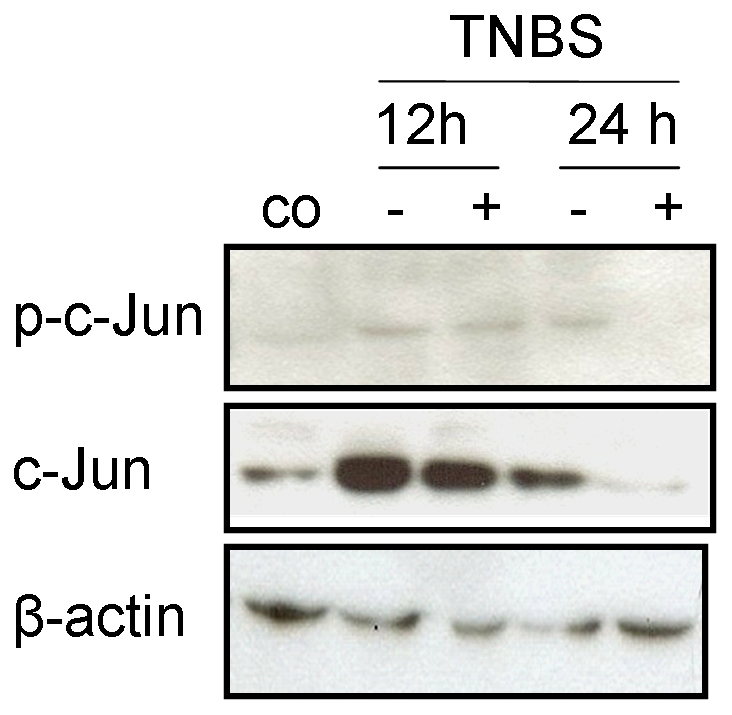
c-Jun and phospho-c-Jun. Western blot analysis of c-Jun and phospho-c-Jun in colon homogenates from untreated controls (co) and 12 h or 24 h following trinitrobenzene sulfonic acid (TNBS) administration without or with XG-102 (100 µg/kg sc.). These blots are representative of 3 independent experiments.

Finally, the expression of c-Jun and its N-terminal phosphorylation exclusively catalyzed by JNKs, were determined either in whole cell extracts by Western blotting (Fig. 8) or immunohistochemistry.

Expression of c-Jun was maximal until 12 h and declined to control levels after 24 h (Fig. 8). Phosphorylation of c-Jun revealed a similar time course. Single application of XG-102 (100 µg/kg sc.) attenuated both the expression and activation of c-Jun (Fig. 8), and this attenuation can be considered as indirect proof of inhibition of JNK by XG-102. Activation of JNK reached its maximum after 3 h and strongly decreased after 12 h; these changes were not affected by XG-102 (data not shown).

In the colon of normal mice, only a restricted number of scattered cells were immunoreactive for c-Jun and immunoreactive phospho-c-Jun was absent. Between 3 h and 12 h after TNBS, the expression of c-Jun increased in the crypts, intestinal wall and infiltrating immune cells, confirming the results from Western blotting (data not shown). The nuclear immunoreactivity of phosphorylated c-Jun was predominantly localized in the damaged epithelium. The intensity of phospho-c-Jun immunoreactivity in infiltrated inflammatory cells correlated with the extent of inflammation. These p-c-Jun signals were substantially attenuated within 12 h following XG-102 (100 µg/kg sc.) (immunohistochemical data not shown).

Thus, the JNK signalling pathway is rapidly activated during early IBD development following local TNBS and reduced by the JNK inhibitor peptide XG-102.

## Discussion

The present study has addressed for the first time the effect of the peptidergic and highly specific JNK inhibitor XG-102 on TNBS- induced acute colon inflammation, a disease model which shares pathophysiological properties of Crohn's disease [38], [39], [42], [43].

The basic and strong pro-inflammatory actions of JNKs and the potent anti-inflammatory effects of JNK-inhibitors [33] raised the question of the protection of inflamed colon tissue by XG-102.

Results indicate that the JNK inhibitor XG-102 protects against characteristic pathological features of TNBS-triggered colon inflammation. The effect of a single application of XG-102 was at least as pronounced or even better as the daily oral gavage of sulfasalazine, a standard drug for experimental and of clinical treatment inflammatory bowel diseases (IBD) [7], [8].

Dose-dependently, XG-102 significantly reduced the clinical parameters (body weight, blood in stool, stool consistency), ulceration, crypt deformations, immune cell infiltration, production of TNF-α, cleavage of caspase-3, expression of Bax, Bim, FasL and p53, the complexation of JNK2 with Bim and finally, expression and activation of c-Jun.

### Normalization of clinical features

A single TNBS instillation induces transient clinical changes (summarized by the disease activity score index, DAI) which return to normal pre-experimental values between 4 and 7 days. In order to detect the protective effects by XG-102, we terminated the experiments after 72 h, i.e. before normalization of DAI components.

In our hands, maximal weight loss reached 14% on average after TNBS, an extent similar to other reports [44], [45]. The action of XG-102 was rather fast, since it significantly attenuated JNK activity and weight loss already after 12 h. In addition, XG-102 confers a long-lasting action at least for 72 h, when the clinical parameters of DAI had returned to pre-experimental values. The re-gain of body weight could be merely due to an improved drinking response, but the normalization of hemoccult and stool consistency indicates a true limitation of the inflammatory process.

### Morphological alterations

The pathogenetic mechanism of TNBS-induced colitis, a delayed-type hypersensitivity response to the hapten-modified self antigens [43], [46], comprises the destruction of epithelial cells and infiltration of lymphocytes, which in turn stimulates a regional inflammation through production of cytokines and other inflammatory mediators [19].

The histological examination of the colon from TNBS-treated mice showed a dramatic inflammation with parameters of immune cell infiltration and ulcerative tissue destruction after 72 h. The JNK inhibitor XG-102 most effectively reduced this pathological histopathology and almost normalized the colon tissue. This is in line with recent studies using different inhibitors of the JNK pathway such as SB203580 or SP600125 [9], [11], [46] or molecules interacting with MAPK like curcumin [47]. For the most part, these inhibitors have been tested in studies of either dextran sulphate sodium–induced (DSS) colitis or hapten-induced colitis (TNBS) in mice or rats, to study their capacities to prevent mucosal inflammation rather than their capacities to reverse established inflammation, a more stringent test.

### Inflammation and immune cell infiltration

XG-102 counteracted the infiltration of immune cells as shown for CD 3- and CD68-positive cells. CD3 is an antigen and part of the T cell receptor (TCR) complex on mature T lymphocytes, and CD68 marks macrophages. This is perfect in line with data from human specimen from IBD patients [20]. The immuno-modulating actions by inhibition of JNK is a common finding in various models of experimental inflammation e.g. animal models for arthritis, pulmonary inflammation, atherosclerosis and other immune challenging procedures (reviewed by [33], [35]).

### Apoptotic and inflammatory features

Activation of caspase-3 is the central event of the major type of cellular apoptosis, and in many cases, it occurs downstream of JNK activation (reviewed by [48]). The almost complete disappearance of cleaved, i.e. activated caspase-3 following XG-102 might be consequence of attenuated Bax or Bim expression as well as the decrease of JNK2:Bim-complex. JNK2, but not JNK1, was found as the JNK isoform responsible for osmotic stress [49]. Features of apoptosis have been regularly described following IBD pathologies including TNBS [39]. The same is true for the production and/or release of TNF-α and the expression of its receptors [11]. Very recently, lamina propria macrophages were identified as a source of TNF-α [50]. Together with other cytokines, TNF-α holds a central position within immune cell activation and tissue destruction, and the application of TNF-α-antibodies and other antagonistic molecules [51] is a novel therapeutic strategy in the treatment of colitis ulcerosa and/or Crohn's disease (reviewed by [7]). However, TNF-α antibody therapy keeps not more than 50% in remission after one year [52], [53], [54].

XG-102 normalizes the enhanced production of TNF-α in colon tissue cultures. JNKs are important mediators of TNF-α signaling [55].

### Mechanisms of XG-102 actions

IBD pathologies rapidly enhance the activation of JNK and its transcriptional effector substrate c-Jun in animals and humans, and link the JNK pathway with the initiation and development of intestinal or gastric inflammation including human IBD [9], [11], [12], [40], [56]. Moreover, a correlation has been proposed between the site of JNK activity and loss of responsiveness to steroids [57].

XG-102 blocks the catalytic action of JNK, but not its activation, by inhibition of JNK scaffold formation [27], [58]. Therefore, the analysis of phosphorylated JNK is inappropriate to visualize the functional level of XG-102. Moreover, antagonisation of JNK activity including inhibitors of the ATP-binding site such as SP600125 increase the level of phosphorylated JNK and their upstream kinases [59] most likely by failure of negative feed back.

The rapid and effective inhibition of JNK by XG-102, i.e. simultaneous but not pre-emptive application with the TNBS provocation, might explain the effectiveness of XG-102. In previous studies with experimental colitis or specimen from patients, inhibition of JNK was followed by down-regulation of c-Jun phosphorylation [9] and AP-1 target genes such as cyclo-oxygenases, MCP-1, ICAM-1 or release of cytokines [9], [20] (reviewed by [21]. Attenuation of activated AP-1 transcription factor c-Jun was seen in experimentally of TNBS and DSS colitis [40], [57]. In our hands, XG-102 attenuates the expression of c-Jun and to a similar extent its N-terminal phosphorylation which is exclusively catalyzed by JNKs.

The recent claim that JNK are not part of the IBD pathology merely derives from a missing rise in JNK expression [60]. Previous observations have already reported on persistent JNK expression with strong rise of its phosphorylation i.e. after cerebral ischemia [61]. JNK are mainly regulated by post-translational modifications and more importantly, by stimulus-specific distribution of JNK isoform within various cellular compartments [61], [62], [63].

The mechanisms of XG-102 are not yet fully understood. Beyond inhibition of JNK functions, it seems to interfere with further pathological processes such as autophagy [64]. The principle of JNK inhibition by peptidergic block of the interaction domain offers the putative advantage to target only JNK signalosoms formed under pathological conditions (discussed [65]. The variation of the length of TAT-sequence and D/L-stereometry enables efficient variations of half life. Moreover, the exclusive specificity of JNK is in striking contrast with the pronounced inhibition of many other kinases by inhibitors of the ATP-domain [66].

The putative use of XG-102 for clinical therapy is tested in clinical studies e.g. of traumatic hearing loss [67] and uveitis ([37]) at present. In pre-clinical animal experiments, XG-102 and a similar peptidergic JNK-inhibitor conferred promising beneficial effects in models for cerebral ischemia ([27], [68], [69]), diabetes ([30], [70]), atherosclerosis ([32]) or lung inflammation (reviewed by [33], [35], [71]).

### Conclusion and outlook

The single application of the JNK inhibitory peptide XG-102 (syn. D-JNK1-I) confers a rapid and lasting anti-inflammatory effectiveness in experimental colitis provoked by the strong immunotoxin TNBS. The therapeutic effect does not require a pre-emptive application nor any latency time. The effects of XG-102 cover a wide range of immunopathological features such as normalization of tissue destruction, loss of body weight and hemorrhage, cytokine production, apoptosis or immune cell invasion.

XG-102 does not directly interfere with the newly identified pathogenic molecules such as NOD2, ATG16L1 or Th17-lymphocytes [5]. Since IBD pathologies are complex multifactorial processes, XG-102 offers the advantage to block a central converging point for several immunopathological signal cascades.

## Materials and Methods

### Ethics Statement

Female BALB/c mice, 7 to 10 weeks old with 16–23 g body weight (Charles River Laboratories, Sulzfeld, Germany), were kept in polycarbonate cages in temperature-controlled rooms with 12 h light/dark cycle and access ad libitum to water and standard rodent food before and during the study. The mice were randomized into groups up to 8 animals. Clinical investigations and all immuno-histological scorings were performed in a double blinded fashion. This study was carried out in strict accordance with the approval of the Ministerium fuer Landwirtschaft und Naturschutz of Schleswig-Holstein (V 312-72241.121-22, 60-6/08 and 74-6/09) and was in agreement with the guidelines for the proper use of animals in biomedical research.

### Induction of colitis by TNBS

Th 1-mediated colitis was induced via rectal instillation of the haptenating agent TNBS (2,4,6-TNBS; Sigma-Aldrich, Munich, Germany). 2 mg TNBS were dissolved in 47.5% ethanol and applied in a final volume of 150 µl per animal. A 3.5-F-catheter was carefully inserted approximately 4 cm into the rectum under short anaesthesia of CO_2_. Mice were carefully held in a vertical position for 1 min after the TNBS administration to ensure distribution of TNBS within the colon and caecum. At the end of all experiments, i.e. between 3 and 72 h after TNBS instillation, the mice were killed by CO_2_ overdose.

### Administration of the test compounds

XG-102 (also termed D-JNKI-1) was synthesized and delivered by Xigen Pharm. (Epalinges, Swiss). XG-102 was dissolved in 0.9% NaCl solution for subcutaneous (sc.) application. XG-102 was tested as 10, 50 and 100 µg/kg for sc. application. XG-102 was given as single dose immediately before TNBS administration. In the TNBS group, the solvent only was injected sc. immediately before rectal TNBS administration.

As positive control, 10 mg/kg sulfasalazine (Sigma-Aldrich, Munich, Germany) was daily applied per os (po) by gavage, the first dose immediately before TNBS instillation. Normal animals served as healthy controls.

### Quantification of disease activity

Disease activity index (DAI) comprising body weight, stool consistency and rectal bleeding was determined daily between 8 and 10 am as described in detail elsewhere [72]. The scores from these three parameters were summed-up as DAI ranging from 0 (healthy) to 12 (maximal severity of colitis).

### Macroscopical inspectation and histological analysis of the colon (H&E stain)

After sacrifice, the colon was quickly removed, gently rinsed with saline solution and the macroscopically visible damage of the colon was assessed. The colon was cut in three parts and tissues were fixed in 4% formalin, embedded in paraffin and cut into 0.5 µm thick sections. Sections were deparaffinized with xylene, rehydrated through graded concentrations of ethanol and stained with hematoxylin (Roth, Karlsruhe, Germany) and eosin (Merck, Darmstadt, Germany) (H&E) using routine techniques. Four to six colon rings from medial and distal colon segment were used for histological examination. Again, histological scoring was performed in a double-blinded manner by two investigators. H&E-scores were determined for 100 µg/kg XG-102 sc. only, and not for the other XG-102 concentrations.

The *inflammation score* was 1, presence of occasional inflammatory cells in the lamina propria; 2, confluence of inflammatory cells extending into the submucosa; 3, transmural extension of the immune cell infiltration.

The *tissue damage score* (ranging from 0 to 9) was composed by the three parameters thickness of submucosa, structure of crypts and erosion/ulcer (each ranging 0–3). Table 1 gives the score details.

**Table 1:** Tissue damage scores.

	Score	parameter
**Submucosa**	0	thickness 0 µm
	1	<15 µm
	2	15–40 µm
	3	>40 µm
**Crypt structure**	0	normal appearance
	1	crypt length reduced by <30%
	2	crypt length reduced by <30%+loss of goblet cells
	3	complete loss of crypts and epithelium
**Erosion/ulceration**	0	epithelium normal, intact
	1	damage of lamina propria
	2	damage of submucosa
3	transmural ulceration	

### Immunofluorescence

7 µm cryosections were fixed in ice-cold acetone (5 min), followed by sequential incubation with blocking solution (normal goat serum, Vector, Burlingame, USA) to eliminate unspecific background staining. Slides were incubated for 1 h at room temperature (RT) with the primary antibody against CD3 (1∶50; rabbit; Abcam, Cambridge, USA) and CD68 (1∶50; rat; Hycult Biotechnology, Beutelsbach, Germany). After washing three times with phosphate-buffered saline (PBS), slides were incubated for 45 min at RT with carbocyanine (Cy3)- or fluorescein isothiocyanate (FITC)-labelled secondary antibody (1∶200; Jackson Immuno Research Lab., Suffolk, UK). Finally, nuclei were counterstained with Finally, nuclei were counterstained with 4′, 6-diamidino-2-phenylindole (Sigma-Aldrich, Munich, Germany).The total number of cells and CD3- or CD68-positive cells were counted in four random sight fields (ROI 600×600 pixels) comprising the mucosa and submucosa each from distal and medial colon segment. The percentages of positive immune cells from the total cell population per vision were calculated. Fluorescence was detected by an Axiophot microscope (LSM510; Zeiss, Jena, Germany) with appropriate filter systems.

### Production of TNF-α

The production of TNF-α was investigated in every second animal (total n = 16). A 1-cm-segment of the transversal colon was removed, cut open longitudinally, and washed in PBS (containing penicillin and streptomycin). The fragment was placed, and incubated in 24 flat-bottom well culture plates containing 1 ml of fresh RPMI 1640 medium (PAA, Cölbe, Germany; supplemented with penicillin and streptomycin) at 37°C for 24 h. TNF-α was quantified using commercially available enzyme-linked immunosorbent assay (ELISA) kits (Cytoset kit; Bio-Source International, Camarillo, USA) according to manufacturers' protocols.

### Preparation of soluble cell lysates

After sacrifice of the animals, the colon tissue was frozen immediately in liquid nitrogen and stored at −80°C. Thereafter, the tissue was homogenized in 0.2–0.4 ml of lysis buffer containing 50 mM Tris-HCl (pH 7.5), 8 mM MgCl_2_, 0.5 mM EDTA, 5 mM EGTA, 250 mM NaCl, 0.01 mg/ml Pepstatin, 0.01 mg/ml Leupeptin, 0.01 mg/ml aprotinin and 1 mM phenylmethylsulphonyl fluoride (Sigma Chemical, St. Louis, USA). The lysates were frozen and thawed three times and incubated on ice for 30 min. After sonication for 10 s, the tissue samples were centrifuged at 15,000× g for 15 min at 4°C. Protein concentrations were determined using Dye Reagent, a variant of Bradford's colorimetric assay (Roth, Karlsruhe, Germany).

### Immunoprecipitation

For immunoprecipitation (IP) native cell lysates were prepared from colon tissue. The tissue was homogenized in native lysis buffer [(20 mM Tris (pH 7.6), 250 mM NaCl, 3 mM EDTA, 3 mM EGTA, 0.5% Non-diet, 1% phosphatase inhibitor (Sigma) and 1% ß-glycerolphosphate and 1% protease inhibitor (Roche)], incubated on ice for 30 min and centrifuged to remove insoluble material (15,000×g for 15 min, 4°C). Supernatants were normalized for total protein content and stored at −80°C. One hundred fifty µg of total proteins were precleared, mixed with the precipitation antibody (1 µg) for 6 h and incubated overnight with 30 µl of 50% of protein-A Sepharose (Sigma). Immobilized protein complexes were washed 3 times with lysis buffer and twice with PBS. The immobilized proteins were dissociated with 5× reducing electrophoresis buffer in 20 µl of ultra-pure water at 95°C for 5 min. the supernatants from the dissociation were carefully loaded onto 15% SDS-gel whereas the matrix was removed by centrifugation.

### Western Blot analysis

Conventional Western blot analysis was conducted as described previously [62]. Primary antibodies against c-Jun (Santa Cruz, Heidelberg, Germany) was diluted 1∶1000, phospho-c-Jun (Cell Signaling Tech., Danvers, USA) 1∶1000, total JNK 1∶1000, phospho-JNK (Promega, Mannheim, Germany) 1∶2000, caspase-3 (Santa Cruz, Heidelberg, Germany) 1∶1000, cleaved caspase-3 (Cell Signaling Tech., Danvers, USA) 1∶1000, Bim 1∶2000, Bax 1∶1000, FasL 1∶1000 (Santa Cruz, Heidelberg, Germany), p53 (Cell Signaling Tech., Danvers, USA) 1∶1000 and against actin (Sigma-Aldrich, Munich, Germany) diluted 1∶5000.

### Immunohistochemistry

Sections of 0.5 µm thickness were deparaffinized in xylene and rehydrated through graded concentrations of ethanol. After rehydration, the endogenous peroxidase was blocked with 2% hydrogen peroxide followed by antigen retrieval by microwaving sections in sodium citrate buffer (10 mM sodium citrate, 0.05% Tween 20, pH 6.0). Following antigen retrieval, the sections were washed with PBS; non-specific binding was blocked with 0.75% bovine serum albumin for 20 min. The incubation with primary antibodies proceeded at 4°C overnight. Antisera against c-Jun (Santa Cruz, Heidelberg, Germany) were diluted 1∶500, phospho-c-Jun (Cell Signaling Tech., Danvers, USA) 1∶250. After repeated washings in PBS, sections were incubated with biotinylated secondary antibody for 45 min followed with an avidin-biotin complex (Vector Laboratories, Burlingame, USA) for 45 min. Controls were processed without the primary antibody. Staining was visualized by diaminobenzidine tetrahydrochloride (Sigma-Aldrich, Munich, Germany) for 5–10 min at RT. The immunohisochemical signals were taken and analysed by a digital camera system (Leica QWin image analysis system, Wetzlar, Germany).

### Statistical analysis

All results including graphics were expressed as the mean + SEM. Statistical analysis was performed by ANOVA 1-way or Kruskal-Wallis test as appropriate followed by Dunnett's t-test or Dunn's test, respectively. The levels of significance *, **, *** were defined as p<0.05, p<0.01 and p<0.001, respectively. The effectiveness of XG-102 and sulfasalazine against TNBS provoked alterations were tested in TNBS animals which received sc. saline.
